# The association between Herpes simplex virus type 2 and asthma: A cross-sectional study from National Health and Nutrition Examination Survey 1999–2016

**DOI:** 10.3389/fmed.2022.943706

**Published:** 2022-09-14

**Authors:** Xiaofei Zhang, Yalin Jiang, Hui Qian, Xiangkun Qu, Kexing Han

**Affiliations:** ^1^Department of Respiratory Medicine, Bozhou Hospital Affiliated to Anhui Medical University, Bozhou, China; ^2^The First Affiliated Hospital of Anhui Medical University, Hefei, China

**Keywords:** Herpes simplex virus type 2, asthma, National Health and Nutrition Examination survey (NHANES), cross-sectional study, prevalence

## Abstract

**Background:**

The association between Herpes simplex virus type 2 (HSV-2) infection, a common infectious disease that increases the incidence of multisystem diseases, and asthma was less well studied. The aim of this study was to investigate the association between HSV-2 infection and the prevalence of asthma.

**Materials and methods:**

We used data from National Health and Nutrition Examination Survey (NHANES) 1999–2016 for analysis. The study population included was limited to those aged 20–45 years and contained complete information on HSV-2 infection and asthma. We calculated the prevalence of HSV-2, asthma, and HSV-2 combined with asthma separately. The association between HSV-2 infection and asthma was analyzed using multiple logistic regression. We also performed stratified analyses to reduce bias and to find sensitive cohorts.

**Results:**

The prevalence of HSV-2 infection was decreasing with change in time period (P for trend < 0.01), but the prevalence of asthma was increasing (P for trend < 0.01). The prevalence of HSV-2 infection was higher in those with asthma than in non-asthma participants. A positive association was found between HSV-2 infection and asthma [odds ratio (OR) = 1.15, 95% CI: 1.04–1.27]. Subgroup analysis showed that this positive association was more pronounced in participants who were male, White, 30 years ≤ age ≤ 40 years, body mass index (BMI) ≤ 28 kg/m^2^, 1.39 ≤ ratio of family income to poverty (PIR) < 3.49 and smokers.

**Conclusion:**

There was a positive association between HSV-2 infection and asthma, and participants who were male, White race, 30 years ≤ age < 40 years, BMI ≥ 28 kg/m^2^, 1.39 ≤ PIR < 3.49, and smokers should receive more attention.

## Introduction

HSV-2 infection is a global health problem that affects an estimated 491.5 million people aged 15–49 years worldwide (1). HSV-2 is transmitted primarily through sexual contact, with herpes genitalis being its early clinical manifestation, and can remain latent in the body for a long time after infection, with the potential for recurrence when the infected person’s immunity is weakened (2). HSV-2 not only affects the urinary and reproductive systems, but also has been shown to cause psychological and social distress (3). Previous studies suggested that HSV-2 infection may lead to reactivation of the virus from a dormant to a proliferative state in the ganglia after a number of triggering factors (e.g., stress, fatigue, heat, cold or ultraviolet radiation, immunosuppression, etc.), resulting in multiple systemic diseases (2). Many researchers have found the presence of this virus in the lung, brain, liver and throat of people infected with HSV-2 (4–7). This suggests that the multisystemic diseases caused by HSV-2 need to be a concern.

According to the 2015 Global Burden of Disease Study, 358 million people are living with asthma worldwide, a 12.6% increase in prevalence from 1990 (8). Bronchial asthma is a heterogeneous disease characterized by chronic airway inflammation and airway hyperresponsiveness, with pathological changes including airway inflammation, airway remodeling and airway hyperresponsiveness. The main etiological causes include genetic and environmental factors, but its pathogenesis has not yet been fully elucidated (9, 10). In non-allergic asthma, viruses are an important factor in causing asthma and contributing to asthma exacerbations. Viral infections cause T-cell and neutrophil-mediated airway inflammation and may be a potential mechanism for non-allergic asthma (11, 12). Banerjee P showed that herpes simplex virus infection affects T-cell-mediated immune responses, for example, patients with herpes virus infection have a lower T1 cell-dominated immune response and an increased T2 cell-dominated immune response, and an enhanced T2 cell-dominated immune response is strongly associated with asthma exacerbations (13). It is also important to note that patients with acute asthma attacks have an increased chance of immunosuppression and this immunosuppressed state increases the patient’s susceptibility to herpes simplex virus (4, 14).

Although many studies have confirmed the association of asthma with viruses, such as rhinovirus (RV) and respiratory syncytial virus (RSV), which are frequently detected in asthma patients (15), studies on the association between herpes simplex virus and asthma were not as well established. Igde M’s study demonstrated a positive correlation between atopic status and HSV-1 infection in children with asthma and allergic rhinitis (16), where the mechanism might be due to the disruption of the body’s immune homeostasis after HSV-1 infection, a view that would be consistent with that of Zhang J (13). To our knowledge, no studies on the association between HSV-2 and asthma have been conducted, probably because HSV-2 testing is extremely private and although patients do not object to testing regarding HSV-2, they may be conservative in feeding back information to healthcare workers (17). Although the relationship between HSV-2 infection and asthma has been studied rarely, HSV-2 infection can cause alterations in the body’s immune response (18), for example, HSV-2 produces a large number of latency-associated transcripts (LATs) during host ganglion latency (19), with five products such as miRNA, sRNA, lncRNA, sncRNA and open reading frames (ORFs). These five products mediate LAT function and play a critical role in HSV-2 reactivation (19), and certain RNAs have been found to be associated with asthma development and asthma severity characteristics, such as hsa-miR-223-3p, a neutrophil-derived microRNA that regulates Toll-like receptors (TLRs)/Th17 signaling and endoplasmic reticulum stress, which causes asthma (20).

Until now, researchers have never stopped exploring the risk factors for asthma. Some studies have shown that the number of genetic loci associated with asthma is continuously increasing, and that changes in many of these loci may be associated with microbial infections, air pollution, and climate change (21). For example, 17q12-21, a genetic locus associated with childhood wheezing asthma, may be strongly associated with a history of viral respiratory infections in early childhood (22). NHANES is a research programme designed to assess the health and nutritional status of the United States population and is often used to conduct cross-sectional studies due to its continuously updated survey data and large sample size. Therefore, we aimed to use data from NHANES 1999–2016 to explore whether there are some notable associations between HSV-2 and asthma.

## Materials and methods

### Data sources

The NHANES database includes participant demographic information, physical examination information, dietary information, and laboratory test information, and has been kept up to date every 2 years since 1999. NHANES project information is open to outside parties, frequently used to conduct cross-sectional studies with large samples, and ethical clearance is exempt.

### Population

**FIGURE 1:**
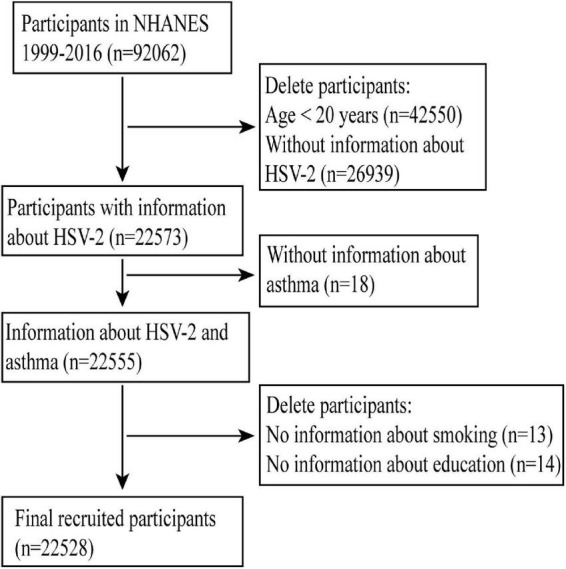
Flow chart for participants.

All participants included in this study participated in the NHANES survey and the age of participants in this study was restricted to 20–49 years of age in accordance with the NHANES regulations for the age of participants who were given a HSV-2 antibody test (the age limit set by this test). All participants included in this study had complete information on HSV-2 antibody test results and whether they had asthma. The study population was screened as shown in Figure 1.

### Dependent and independent variable

In this study, we explored the association between HSV-2 infection and asthma by using whether the participants were infected with HSV-2 as the independent variable and whether they had asthma as the dependent variable.

A subsample of serum for the presence of glycoprotein-specific HSV-2 (designated as gG-2) was detected using a highly sensitive and specific solid-phase enzymatic type-specific immunospot assay, and a positive antibody was defined as an infection. NHANES 1999–2016 laboratory data section contained complete information on herpes simplex virus antibody testing.

The NHANES 1999–2016 questionnaire included information on medical conditions and participants were asked the question “Have you ever been told have asthma?” Participants who answered “Yes” were defined as asthma sufferers.

### Covariates

Based on previous studies (23), we collected age, gender, race, BMI, education, marital status, total household size, PIR, smoking status, alcohol use, sexual behavior, injecting drug use, and as potential influencing factors, and the presence of a close relative with asthma as an influencing factor for asthma. Smoking status, alcohol consumption, sexual behavior and injecting drug use were taken from the questionnaire section. Smokers were defined as having smoked 100 cigarettes in their lifetime, and drinking 12 times in the past year was defined as drinking alcohol. Information on sexual behavior and injecting drug use was obtained from participants’ responses to the questions “Ever had vaginal, anal, or oral sex?” and “Ever use a needle to inject illegal drug?”

### Statistical analysis

All data extraction and analyses were performed in R^1^ and Empowerstats.^2^ To make the included information more representative of the entire United States population, we used 2-year sample weights throughout the analyses. Prevalence rates were expressed using weighted rates with 95% confidence intervals (95% CI), while trend tests for rates were conducted on a year-by-year basis. In the cleaning process of the covariate data, we made some allowance for missing data. When the missing data were continuous variables and did not exceed 10% of the total sample, we used the mean as a substitution. When the missing variable was categorical, we defined it as a group that could be independently classified (Unclear group) if the sample size exceeded 10, otherwise it was removed. Multiple logistic regression was used to analyze the relationship between HSV-2 and asthma. Three models were generated by adjusting for different covariates in order to more fully assess the relationship between the independent and response variables. Model 1: non-adjusted model; Model 2: age, gender, and race were adjusted; Model 3: all covariates presented in Table 1 were adjusted. To find sensitive cohorts, we then conducted a stratified analysis. We set a *p*-value < 0.05 as statistically different.

**TABLE 1:** Baseline characteristics of included participants.

Characteristic	Without asthma	Asthma	*P*-value
Sample size	19364	3164	
HSV-2 infection (%)			< 0.001
Yes	20.77	25.19	
No	79.23	74.81	
Gender (%)			< 0.001
Male	47.91	40.71	
Female	52.09	59.29	
Age (years)	34.50 ± 8.62	33.23 ± 8.72	< 0.001
Stratified by age (years) (%)			< 0.001
< 30	32.94	39.79	
30–40	33.31	31.64	
≥ 30	33.75	28.57	
Race (%)			< 0.001
Mexican American	22.38	10.49	
White	39.76	48.39	
Black	19.92	24.02	
Other race	17.94	17.10	
Marital status (%)			< 0.001
Live with a partner	61.61	52.88	
Solitude	37.25	46.40	
Unclear	1.13	0.73	
Family members (%)			< 0.001
< 3	45.79	52.50	
≥ 3	54.21	47.50	
BMI (kg/m^2^)	28.46 ± 6.80	29.99 ± 8.02	< 0.001
Stratified by BMI (kg/m^2^) (%)			< 0.001
< 24	26.80	23.32	
24–27.9	27.14	23.93	
≥ 28	46.05	52.75	
PIR (%)	2.49 ± 1.57	2.37 ± 1.61	< 0.001
Stratified by PIR (%)			< 0.001
< 1.39	32.19	37.33	
1.39–3.49	38.97	34.83	
≥ 3.49	28.84	27.84	
Education (%)			< 0.001
<High school	23.92	18.39	
High school	23.04	21.65	
> High school	53.05	59.96	
Smoker (%)			< 0.001
Yes	39.85	46.21	
No	60.15	53.79	
Alcohol drinking (%)			0.004
Yes	11.69	11.44	
No	11.96	9.99	
Unclear	76.35	78.57	
Sexual intercourse (%)			< 0.001
Yes	84.06	87.55	
No	4.88	3.82	
Unclear	11.07	8.63	
Used injecting drugs (%)			< 0.001
Yes	1.54	2.28	
No	65.46	71.27	
Unclear	33.00	26.45	
Close relatives with asthma (%)			< 0.001
Yes	19.47	45.48	
No	79.01	51.58	
Unclear	1.52	2.94	

Mean + SD for continuous variables: P-value was calculated by weighted linear regression model. % for Categorical variables: P-value was calculated by weighted Chi-square test. HSV-2, Herpes simplex virus type 2; BMI, Body mass index (kg/m^2^); PIR, Ratio of family income to poverty.

## Results

### Prevalence of Herpes simplex virus type 2 and asthma

**FIGURE 2:**
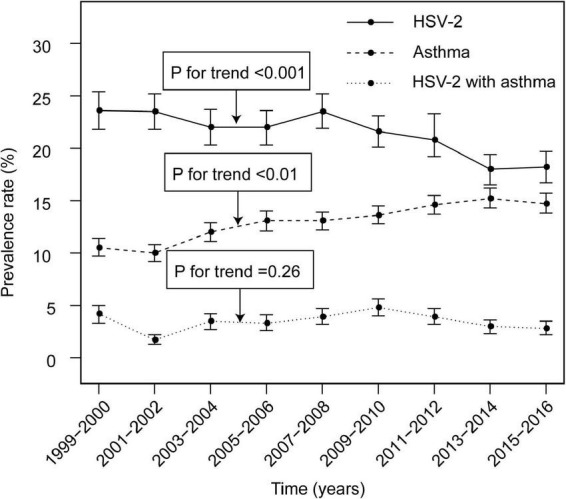
Prevalence and trends of HSV-2 infection and asthma. HSV-2, Herpes simplex virus type 2.

NHANES updates data from the survey on a 2-year time period. We calculated prevalence rates for HSV-2, asthma and co-morbidities for 9 time periods in NHANES 1999–2016 and tested for trends in these prevalence rates over the time period. The prevalence of HSV-2 was 21.4% (4825/22573), (95% CI: 20.8–21.9%) during 1999–2016, with a trend test suggested a decreasing trend in the prevalence of HSV-2 over time (P for trend < 0.01). The prevalence of asthma was 13.1% (6457/49463), (95% CI: 12.8–13.4%), but the prevalence was increasing (P for trend < 0.01). The prevalence of co-existing HSV-2 and asthma was 3.4% (798/23555), (95% CI: 3.2–3.6%), and the trend test for the rate was not statistically different (P for trend = 0.26). Results are presented in Supplementary Tables 1–3 and Figure 2.

### Baseline characteristics of study participants

A total of 22,528 participants were eventually included, of whom 3,164 were asthmatic and 4,819 were HSV-2 antibody positive. A higher proportion of participants with asthma had HSV-2 infection than non-asthmatic participants (*p* < 0.01) (Table 1).

### The association between Herpes simplex virus type 2 and asthma

**FIGURE 3:**
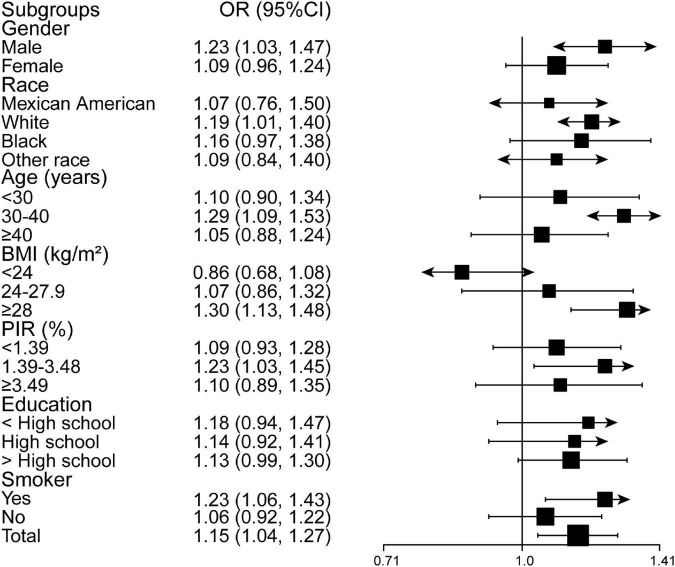
Subgroups analyses of the association between HSV-2 and asthma. HSV-2, Herpes simplex virus type 2. All the covariates in Table 1 were adjusted.

There was a positive association between HSV-2 and asthma (OR = 1.15, 95% CI: 1.04–1.27) (Table 2). Stratified analyses suggested that sensitive individuals with statistical differences were male (OR = 1.23, 95% CI: 1.03–1.47), White race (OR = 1.19, 95% CI: 1.01–1.40), 30 ≤ age (years) < 40 (OR = 1.29, 95% CI: 1.09–1.53), BMI ≥ 28 kg/m^2^ (OR = 1.30, 95% CI: 1.13–1.48), 1.39 ≤ PIR < 3.49 (OR = 1.23, 95% CI: 1.03, 1.45), and smoking participants (OR = 1.23, 95% CI: 1.06–1.43). In addition, this positive relationship was more pronounced for those with education below high school level than for other education levels (OR = 1.18, 95% CI: 0.94–1.47). The sensitive population in the fully adjusted model condition is shown in Figure 3.

**TABLE 2:** Association between HSV-2 infection and asthma.

Characteristic	Model 1, OR (95% CI)	Model 2, OR (95% CI)	Model 3, OR (95% CI)
HSV-2 (–)	1.0	1.0	1.0
HSV-2 (+)	1.28 (1.18, 1.40)	1.27 (1.15, 1.40)	1.15 (1.04, 1.27)
Subgroups			
Gender			
Male	1.13 (0.97, 1.33)	1.28 (1.08, 1.52)	1.23 (1.03, 1.47)
Female	1.28 (1.15, 1.42)	1.26 (1.12, 1.42)	1.09 (0.96, 1.24)
Race			
Mexican American	1.10 (0.81, 1.51)	1.18 (0.85, 1.63)	1.07 (0.76, 1.50)
White	1.28 (1.10, 1.48)	1.33 (1.15, 1.55)	1.19 (1.01, 1.40)
Black	1.12 (0.96, 1.31)	1.16 (0.99, 1.38)	1.16 (0.97, 1.38)
Other race	1.34 (1.08, 1.68)	1.42 (1.13, 1.79)	1.09 (0.84, 1.40)
Age (years)			
< 30	1.27 (1.07, 1.52)	1.15 (0.95, 1.39)	1.10 (0.90, 1.34)
30 ≤ age < 40	1.50 (1.29, 1.74)	1.39 (1.18, 1.64)	1.29 (1.09, 1.53)
≥ 40	1.38 (1.20, 1.60)	1.22 (1.04, 1.43)	1.05 (0.88, 1.24)
BMI (kg/m^2^)			
< 24	0.93 (0.75, 1.15)	0.94 (0.75, 1.18)	0.86 (0.68, 1.08)
24–27.9	1.15 (0.96, 1.39)	1.18 (0.96, 1.44)	1.07 (0.86, 1.32)
≥ 28	1.42 (1.27, 1.60)	1.38 (1.21, 1.57)	1.30 (1.13, 1.48)
PIR (%)			
< 1.39	1.33 (1.16, 1.53)	1.20 (1.03, 1.40)	1.09 (0.93, 1.28)
1.39–3.49	1.29 (1.12, 1.50)	1.29 (1.10, 1.52)	1.23 (1.03, 1.45)
≥ 3.49	1.12 (0.93, 1.35)	1.15 (0.94, 1.41)	1.10 (0.89, 1.35)
Education			
< High school	1.61 (1.34, 1.93)	1.32 (1.07, 1.62)	1.18 (0.94, 1.47)
High school	1.22 (1.02, 1.47)	1.25 (1.02, 1.53)	1.14 (0.92, 1.41)
> High school	1.26 (1.12, 1.42)	1.29 (1.13, 1.47)	1.13 (0.99, 1.30)
Smoker			
Yes	1.24 (1.10, 1.41)	1.26 (1.09, 1.45)	1.23 (1.06, 1.43)
No	1.25 (1.11, 1.42)	1.15 (1.00, 1.31)	1.06 (0.92, 1.22)

Model 1: No covariates were adjusted. Model 2: Age, gender, race were adjusted. Model 3: Age, gender, race, body mass index, poverty to income ratio, education, marital status, family members, smoker, alcohol drinking, sexual intercourse, used injecting drugs, close relatives with asthma were adjusted. The model is not adjusted for the stratification variable itself in the subgroup analysis. HSV-2, Herpes simplex virus type 2; BMI, Body mass index (kg/m^2^); PIR, Ratio of family income to poverty.

## Discussion

In this study, we first assessed changes in the prevalence of HSV-2, asthma and co-morbidities of both in the United States from 1999–2016. Similar to the results of previous studies, the prevalence of HSV-2 infection showed a decreasing trend over time, which is closely related to past public health efforts (24–26). For asthma, smoking, environmental pollution and climate change may be potential reasons for the continued increase in asthma prevalence (8). In addition, we demonstrated a positive association between HSV-2 and asthma (OR = 1.15, 95% CI: 1.04–1.27) and identified sensitive cohorts.

Although the study by Zein et al. found that the prevalence of asthma would shift from males to females after adolescence (27), the stratified analysis in this study suggested that the positive association between HSV-2 infection and asthma was more pronounced in the male population, and that this positive association may exist because of the higher proportion of males who smoke, which has become known to be a significant factor that can contribute to asthma (28). Neither asthma nor HSV-2 infection appears to be prevalent in populations of white ethnicity (29, 30). However, on the basis of the positive association between the two diseases suggested by this study, White race were more sensitive. Combined with the results of the baseline patient data in Table 1, the proportion of patients with asthma in this study was indeed higher for the White race. Although there are still no larger cohort studies that have found similar results, whether HSV-2 infection exhibits a more severe Th2 response (11, 31) and thus causes asthma can explain the greater susceptibility to asthma after HSV-2 infection in White race compared to other races remains to be further validated by subsequent studies.

A study based on the National Center for Health Statistics (NCHS) confirmed that the prevalence of HSV-2 increases with age (age range 14–49 years), with a positive rate of up to 21.2% in the 40–49 age group (30). Although the age cut-off for late-onset asthma is still controversial, the prevailing view is that 50 years is a reasonable value (32). The proportion of the cohort with late-onset asthma is clearly smaller compared to other age groups, and evidence from epidemiological studies has shown that the proportion of patients with asthma decreases progressively with age. This may be the reason why our study found that the positive association between HSV-2 infection and asthma was more pronounced in participants aged 30–39 years.

In our study, we found that the positive association between HSV-2 infection and asthma was more pronounced in obese participants. There is no denying that obesity is closely associated with asthma and that obese asthma patients have more severe clinical symptoms and a poorer quality of life (33). Asthma as a chronic disease imposes a significant financial burden on society and individuals (34), and although children as a high prevalence group of asthma place greater socio-economic pressure, studies have shown that the average cost to individuals for managing asthma increases with age, and for groups with very low financial capacity may choose to discontinue treatment (35). This might explain why the positive association between HSV-2 infection and asthma in our study was more pronounced in participants of moderate financial capacity. Although the stratified analysis in this study did not yield statistically significant differences between the different education levels, the effect sizes of the regression models could somewhat suggest that the positive association between HSV-2 and asthma might improve with increasing education level.

Although cross-sectional studies cannot be used as evidence to explain causality, to our knowledge, our study is the first to explore the relationship between HSV-2 infection and asthma. Although not all cohorts were statistically significant in the stratified analysis, the trend toward a positive association between HSV-2 infection and asthma was stable on the basis of adjustment for different covariates.

However, there were also some limitations to our study. For example, risk factors for asthma are still not fully identified and we could not ensure that the full range of confounding factors were included as covariates in the adjustment. Secondly, due to the characteristics of the available data in NHANES, the diagnosis of asthma in this study was based on participants’ responses to a questionnaire, and it might have been difficult for us to discriminate between participants with non-allergic and allergic asthma and to grade their disease severity based on ancillary tests. To further explore the correlation between HSV-2 and asthma, follow-up studies will need to be supplemented with more complete information. In addition, cross-sectional studies are naturally unavoidable in terms of missing variables, and although we have treated missing variables accordingly, the impact of missing variables on the final results might still be difficult to avoid, so conclusions from cross-sectional studies such as these need to be treated with caution. Finally, due to the presence of missing variables, we did not stratify the characteristics of participants with missing variables in order to avoid biasing the results of the stratification analysis by missing variables and to limit our study from further identifying potential special populations of interest.

## Conclusion

A positive correlation existed between HSV-2 infection and asthma. While the trend of HSV-2 infection decreased over time, the prevalence of asthma was on the rise. Male, white race, age 30–40 years, weight ≥ 28 kg/m^2^, 1.39 ≤ PIR < 3.49, smokers should be more cautious in the positive association between HSV-2 infection and asthma.

## Data availability statement

The original contributions presented in this study are included in the article/Supplementary material, further inquiries can be directed to the corresponding author.

## Author contributions

XZ: data analysis. XQ: manuscript writing. HQ and YJ: manuscript editing. KH: study design and quality control. All authors agreed on the journal to which the article was to be submitted and agreed to take responsibility for all aspects of the work.

## Footnotes

1
http://www.R-project.org

2
http://www.empowerstats.com

## References

[1] James C Harfouche M Welton NJ Turner KM Abu-Raddad LJ Gottlieb SL Herpes simplex virus: global infection prevalence and incidence estimates, 2016. *Bull World Health Organ.* (2020). 98:315–29. 10.2471/BLT.19.237149 32514197 PMC7265941

[2] Whitley RJ Roizman B. Herpes simplex virus infections. *Lancet.* (2001) 357:1513–8. 10.1016/S0140-6736(00)04638-9 11377626

[3] Gupta R Warren T Wald A. Genital herpes. *Lancet.* (2007) 370:2127–37. 10.1016/S0140-6736(07)61908-4 18156035

[4] Gasparetto EL Escuissato DL Inoue C Marchiori E Müller NL. Herpes simplex virus type 2 pneumonia after bone marrow transplantation: high-resolution CT findings in 3 patients. *J Thorac Imaging.* (2005) 20:71–3. 10.1097/01.rti.0000154072.39497.61 15818204

[5] Corsini Campioli C Abu Saleh O. Herpes simplex virus as a cause of pharyngitis. *Lancet Infect Dis.* (2020) 20:628. 10.1016/S1473-3099(20)30018-9 32359469

[6] Lai V Meher-Homji Z Liu F Sasadeusz J. Primary herpes simplex virus type 2 hepatitis diagnosed during laparoscopy. *Lancet.* (2020) 396:e90. 10.1016/S0140-6736(20)32388-6 33278939

[7] Armangue T Spatola M Vlagea A Mattozzi S Cárceles-Cordon M Martinez-Heras E Frequency, symptoms, risk factors, and outcomes of autoimmune encephalitis after herpes simplex encephalitis: a prospective observational study and retrospective analysis. *Lancet Neurol.* (2018) 17:760–72. 10.1016/S1474-4422(18)30244-8 30049614 PMC6128696

[8] Gbd 2015 Chronic Respiratory Disease Collaborators. Global, regional, and national deaths, prevalence, disability-adjusted life years, and years lived with disability for chronic obstructive pulmonary disease and asthma, 1990–2015: a systematic analysis for the global burden of disease study 2015. *Lancet Respir Med.* (2017) 5:691–706. 10.1016/S2213-2600(17)30293-X 28822787 PMC5573769

[9] Sockrider M Fussner L. What is asthma. *Am J Respir Crit Care Med.* (2020) 202:25–6. 10.1164/rccm.2029P25 33124914

[10] Halayko AJ Pascoe CD Gereige JD Peters MC Cohen RT Woodruff PG. Update in adult asthma 2020. *Am J Respir Crit Care Med.* (2021) 204:395–402. 10.1164/rccm.202103-0552UP 34181860 PMC8759264

[11] Hammad H Lambrecht BN. The basic immunology of asthma. *Cell.* (2021) 184:1469–85. 10.1016/j.cell.2021.02.016 33711259

[12] Castillo JR Peters SP Busse WW. Asthma exacerbations: pathogenesis, prevention, and treatment. *J Allergy Clin Immunol Pract.* (2017) 5:918–27. 10.1016/j.jaip.2017.05.001 28689842 PMC5950727

[13] Zhang J Liu H Wei B. Immune response of T cells during herpes simplex virus type 1 (HSV-1) infection. *J Zhejiang Univ Sci B.* (2017) 18:277–88. 10.1631/jzus.B1600460 28378566 PMC5394093

[14] Banerjee P Balraj P Ambhore NS Wicher SA Britt RD Jr Pabelick CM Network and co-expression analysis of airway smooth muscle cell transcriptome delineates potential gene signatures in asthma. *Sci Rep.* (2021) 11:14386. 10.1038/s41598-021-93845-x 34257337 PMC8277837

[15] Jartti T Bønnelykke K Elenius V Feleszko W. Role of viruses in asthma. *Semin Immunopathol.* (2020) 42:61–74. 10.1007/s00281-020-00781-5 31989228 PMC7066101

[16] Igde M Igde FA Yazici Z. Herpes simplex type I infection and atopy association in Turkish children with asthma and allergic rhinitis. *Iran J Allergy Asthma Immunol.* (2009) 8:149–54. 20124606

[17] Cham HJ Lasswell SM Miller KS. Parents’ reactions to testing for herpes simplex virus type 2 as a biomarker of sexual activity in Botswana junior secondary school students. *Sex Health.* (2016) 13:148–54. 10.1071/SH15092 26886026 PMC4966994

[18] Evans IA Jones CA. HSV induces an early primary Th1 CD4 T cell response in neonatal mice, but reduced CTL activity at the time of the peak adult response. *Eur J Immunol.* (2005) 35:1454–62. 10.1002/eji.200425333 15789359

[19] Zhang Y Xin Q Zhang JY Wang YY Cheng JT Cai WQ Transcriptional regulation of latency-associated transcripts (LATs) of herpes simplex viruses. *J Cancer.* (2020) 11:3387–99. 10.7150/jca.40186 32231745 PMC7097949

[20] Gomez JL Chen A Diaz MP Zirn N Gupta A Britto C A network of sputum microRNAs is associated with neutrophilic airway inflammation in asthma. *Am J Respir Crit Care Med.* (2020) 202:51–64. 10.1164/rccm.201912-2360OC 32255668 PMC7328332

[21] El-Husseini ZW Gosens R Dekker F Koppelman GH. The genetics of asthma and the promise of genomics-guided drug target discovery. *Lancet Respir Med.* (2020) 8:1045–56. 10.1016/S2213-2600(20)30363-5 32910899

[22] Koppelman GH Kersten E. Understanding how asthma starts: longitudinal patterns of wheeze and the chromosome 17q locus. *Am J Respir Crit Care Med.* (2021) 203:793–5. 10.1164/rccm.202102-0443ED 33621469 PMC8017592

[23] Zhang X Xu Y Li Y Yuan H Liu Z Zhang T. Prevalence and correlates of Kaposi’s sarcoma-associated herpesvirus and herpes simplex virus type 2 infections among adults: evidence from the NHANES III data. *Virol J.* (2022) 19:5. 10.1186/s12985-021-01731-9 34991626 PMC8740377

[24] Shangase N Kharsany A Ntombela NP Pettifor A McKinnon LRA. Systematic review of randomized controlled trials of school based interventions on sexual risk behaviors and sexually transmitted infections among young adolescents in sub-Saharan Africa. *AIDS Behav.* (2021) 25:3669–86. 10.1007/s10461-021-03242-8 33772695 PMC8985547

[25] Chemaitelly H Nagelkerke N Omori R Abu-Raddad LJ. Characterizing herpes simplex virus type 1 and type 2 seroprevalence declines and epidemiological association in the United States. *PLoS One.* (2019) 14:e0214151. 10.1371/journal.pone.0214151 31170140 PMC6553692

[26] Wald A Langenberg AG Krantz E Douglas JM Jr Handsfield HH DiCarlo RP The relationship between condom use and herpes simplex virus acquisition. *Ann Intern Med.* (2005) 143:707–13. 10.7326/0003-4819-143-10-200511150-00007 16287791

[27] Zein JG Erzurum SC. Asthma is different in women. *Curr Allergy Asthma Rep.* (2015) 15:28. 10.1007/s11882-015-0528-y 26141573 PMC4572514

[28] Çolak Y Afzal S Nordestgaard BG Lange P. Characteristics and prognosis of never-smokers and smokers with asthma in the Copenhagen general population study. A prospective cohort study. *Am J Respir Crit Care Med.* (2015) 192:172–81. 10.1164/rccm.201502-0302OC 25914942

[29] Flores C Ma SF Pino-Yanes M Wade MS Pérez-Méndez L Kittles RA African ancestry is associated with asthma risk in African Americans. *PLoS One.* (2012) 7:e26807. 10.1371/journal.pone.0026807 22235241 PMC3250386

[30] McQuillan G Kruszon-Moran D Flagg EW Paulose-Ram R. Prevalence of herpes simplex virus type 1 and type 2 in persons aged 14-49: United States, 2015–2016. *NCHS Data Brief.* (2018) 304:1–8. 29442994

[31] Nakajima H Kobayashi M Pollard RB Suzuki F. A pathogenic role of Th2 responses on the severity of encephalomyelitis induced in mice by herpes simplex virus type 2 infection. *J Neuroimmunol.* (2000) 110:106–13. 10.1016/s0165-5728(00)00353-2 11024539

[32] Stern J Pier J Litonjua AA. Asthma epidemiology and risk factors. *Semin Immunopathol.* (2020) 42:5–15. 10.1007/s00281-020-00785-1 32020334

[33] Toskala E Kennedy DW. Asthma risk factors. *Int Forum Allergy Rhinol.* (2015) 5(Suppl. 1):S11–6. 10.1002/alr.21557 26335830 PMC7159773

[34] Szefler SJ Zeiger RS Haselkorn T Mink DR Kamath TV Fish JE Economic burden of impairment in children with severe or difficult-to-treat asthma. *Ann Allergy Asthma Immunol.* (2011) 107:110–9.e1. 10.1016/j.anai.2011.04.008 21802018

[35] Lee YH Yoon SJ Kim EJ Kim YA Seo HY Oh IH. Economic burden of asthma in Korea. *Allergy Asthma Proc.* (2011) 32:35–40. 10.2500/aap.2011.32.3479 22221428

