# Supplementary material for: The association between Herpes simplex virus type 2 and asthma: A cross-sectional study from National Health and Nutrition Examination Survey 1999–2016
Source: Front Med (Lausanne). 2022 Sep 14;9:943706. doi: 10.3389/fmed.2022.943706 (PMC9515305; doi:10.3389/fmed.2022.943706)
Supplement: Supplementary file 1 [file Data_Sheet_1.doc]

**Supplementary table 1.** Prevalence of HSV-2 infection from NHANES 1999-2016.

| Time (years) | HSV-2 (+) | HSV-2 (-) | Adjusted%, 95%CI |
| --- | --- | --- | --- |
| 1999-2000 | 493 | 1594 | 23.6, (21.8, 25.4) |
| 2001-2002 | 590 | 1920 | 23.5, (21.8, 25.2) |
| 2003-2004 | 490 | 1737 | 22.0, (20.3, 23.7) |
| 2005-2006 | 539 | 1914 | 22.0, (20.3, 23.6) |
| 2007-2008 | 597 | 1941 | 23.5, (21.9, 25.2) |
| 2009-2010 | 629 | 2283 | 21.6, (20.1, 23.1) |
| 2011-2012 | 526 | 2008 | 20.8, (19.2, 23.3) |
| 2013-2014 | 491 | 2242 | 18.0, (16.5, 19.4) |
| 2015-2016 | 470 | 2109 | 18.2, (16.7, 19.7) |
| Over all | 4825 | 17748 | 21.4, (20.8, 21.9) |
| P-value for trend<0.001 | | | |

Abbreviation: HSV-2, Herpes simplex virus type 2; NHANES: National Health and Nutrition Examination Survey.

**Supplementary table 2.** Prevalence of asthma from NHANES 1999-2016.

| Time (years) | With asthma | Without asthma | Adjusted%, 95%CI |
| --- | --- | --- | --- |
| 1999-2000 | 514 | 4361 | 10.5, (9.7, 11.4) |
| 2001-2002 | 540 | 4867 | 10.0, (9.2, 10.8) |
| 2003-2004 | 602 | 4432 | 12.0, (11.1, 12.9) |
| 2005-2006 | 650 | 4322 | 13.1, (12.1, 14.0) |
| 2007-2008 | 774 | 5155 | 13.1, (12.2, 13.9) |
| 2009-2010 | 847 | 5364 | 13.6, (12.8, 14.5) |
| 2011-2012 | 810 | 4746 | 14.6, (13.7, 15.5) |
| 2013-2014 | 878 | 4886 | 15.2, (14.3, 16.2) |
| 2015-2016 | 842 | 4873 | 14.7, (13.8, 15.7) |
| Over all | 6457 | 43006 | 13.1, (12.8, 13.4) |
| *P*-value for trend<0.01 | | | |

Abbreviation: NHANES: National Health and Nutrition Examination Survey.

**Supplementary table 3.** Prevalence of participants with combined HSV-2 infection and asthma from NHANES 1999-2016.

| Time (years) | HSV-2 with asthma | HSV-2 without asthma | Adjusted%, 95%CI |
| --- | --- | --- | --- |
| 1999-2000 | 87 | 2000 | 4.2, (3.3, 5.0) |
| 2001-2002 | 61 | 3448 | 1.7, (1.3, 2.2) |
| 2003-2004 | 77 | 2147 | 3.5, (2.7, 4.2) |
| 2005-2006 | 82 | 2369 | 3.3, (2.6, 4.1) |
| 2007-2008 | 99 | 2435 | 3.9, (3.2, 4.7) |
| 2009-2010 | 139 | 2769 | 4.8, (4.0, 5.6) |
| 2011-2012 | 99 | 2435 | 3.9, (3.2, 4.7) |
| 2013-2014 | 81 | 2649 | 3.0, (2.3, 3.6) |
| 2015-2016 | 73 | 2505 | 2.8, (2.2, 3.5) |
| Over all | 798 | 22757 | 3.4, (3.2, 3.6) |
| *P*-value for trend=0.26 | | | |

Abbreviation: HSV-2, Herpes simplex virus type 2; NHANES: National Health and Nutrition Examination Survey.
